# Structural and Electrical Properties of Annealed Ge_2_Sb_2_Te_5_ Films Grown on Flexible Polyimide

**DOI:** 10.3390/nano12122001

**Published:** 2022-06-10

**Authors:** Marco Bertelli, Adriano Díaz Fattorini, Sara De Simone, Sabrina Calvi, Riccardo Plebani, Valentina Mussi, Fabrizio Arciprete, Raffaella Calarco, Massimo Longo

**Affiliations:** 1 Institute for Microelectronics and Microsystems (IMM), Consiglio Nazionale delle Ricerche (CNR), Via del Fosso del Cavaliere 100, 00133 Rome, Italy; marco.bertelli@artov.imm.cnr.it (M.B.); adriano.diazfattorini@artov.imm.cnr.it (A.D.F.); sara.desimone@artov.imm.cnr.it (S.D.S.); valentina.mussi@artov.imm.cnr.it (V.M.); massimo.longo@artov.imm.cnr.it (M.L.); 2 Department of Physics, University of “Tor Vergata”, Via della Ricerca Scientifica 1, 00133 Rome, Italy; sabrina.calvi@roma2.infn.it (S.C.); plebaniriccardo@gmail.com (R.P.); fabrizio.arciprete@roma2.infn.it (F.A.)

**Keywords:** PCM, Ge_2_Sb_2_Te_5_, sputtering, flexible substrates, crystallization, electrical properties

## Abstract

The morphological, structural, and electrical properties of as-grown and annealed Ge_2_Sb_2_Te_5_ (GST) layers, deposited by RF-sputtering on flexible polyimide, were studied by means of optical microscopy, atomic force microscopy, X-ray diffraction, Raman spectroscopy, and electrical characterization. The X-ray diffraction annealing experiments showed the structural transformation of GST layers from the as-grown amorphous state into their crystalline cubic and trigonal phases. The onset of crystallization of the GST films was inferred at about 140 °C. The vibrational properties of the crystalline GST layers were investigated via Raman spectroscopy with mode assignment in agreement with previous works on GST films grown on rigid substrates. The electrical characterization revealed a good homogeneity of the amorphous and crystalline trigonal GST with an electrical resistance contrast of 8 × 10^6^.

## 1. Introduction

Tremendous advances in interconnected technologies are already revolutionizing our lives by integrating networks of physical devices (‘things’) that are capable of capturing, processing, and sharing data over the internet (Internet of Things (IoT)). Neuromorphic computing based on innovative concepts inspired by the functioning or organization of the brain’s data processing chain provides answers to such challenges [1].

Revolutionary advances in computing architecture require different methods of managing computing memory and the development of alternative technologies for its implementation. This can be realized in practice by exploiting the physical properties of devices that show a certain degree of time nonlocality (memory) in their response functions, such as memristors [2].

Synaptic connections have been realized with different types of memristors [3,4,5], among others, with phase change memories (PCMs) [6,7], being extensively used in the semiconductor industry [8]. PCMs are based on chalcogenide alloys that are able to reversibly change their resistance upon the application of proper electrical stimuli. The resistance change exploits the different resistivities of two distinct structural solid-state phases (i.e., crystalline and amorphous), while the switching mechanism is thermally induced through Joule heating [9].

The deposition of PCMs on flexible substrates may have a huge impact on the market of electronic applications, for example, flexible nonvolatile memories for the IoT [10] or smart sensors for food and drug monitoring [11].

Polyimide (PI) is one of the most interesting candidates as a flexible substrate for PCM deposition because it is insulating (electrical resistivity *ρ* = 0.1–4 × 10^19^ Ωcm), lightweight (density *d* = 0.005–1.88 g/cm^3^), and resistant to heat (in air maximum operating temperature T_max_ = 200–395 °C) [12]. PI is already being used in the electronic industry to realize flexible printed circuits [13]. Moreover, the possibility to produce transparent and/or biocompatible PI films [14,15] makes them extremely attractive for wearable smart devices and biosensors.

In previous works, we have studied the synthesis and the crystallization of single Ge-Sb-Te films with variable Ge content [16], as well as heterostructures formed by planar layers of the Ge-Sb-Te system [17]. Moreover, the self-assembly and structural characterization of core-shell nanowires of the Ge-Sb-Te system was investigated [18].

In the present work, amorphous GST films were deposited by RF-sputtering on flexible PI. The layers were studied in terms of their morphological, structural, and electrical properties prior to and after crystallization using dedicated thermal annealing during structural investigation. While a few studies have already examined the growth of GST layers on flexible substrates (i.e., mica [19], polycarbonate [20], and PI [21,22,23,24,25,26]), we could not find a systematic study on GST/PI structures reporting the morphological, structural, vibrational, and electrical properties of as-deposited (amorphous) and annealed (crystalline) thin (≤150 nm) GST layers. We obtained information about the layer evolution during the amorphous-to-cubic and cubic-to-trigonal transitions and the related electrical contrast. This is important for the functioning of PCM devices based on a reversible switch between material phases and the association of binary encoding to the consequent resistivity changes. Therefore, the implementation of such stacks in flexible memristive devices should provide further advantages in terms of memristor performance (e.g., reduction in the switching energy and higher storage density).

## 2. Materials and Methods

### 2.1. Samples Growth

The GST layers were deposited by RF-sputtering in a custom-made high-vacuum chamber system (IONVAC PROCESS srl, Pomezia, Italy) equipped with a planetary system for deposition and four confocal targets. GST films (thickness ~140 nm) were grown at room temperature (RT) on flexible PI (DuPont™ Kapton^®^ HN, thickness = 125 µm, Wilmington, DE, USA), which was coated with a protective silicon-nitride layer (thickness ~200 nm). The GST target was provided by Robeko GmbH & Co., KG (Mehlingen, Germany), with a nominal composition of Ge_2_Sb_2_Te_5_ with 99.99% purity. The sputtering forward power was 50 W, and the flow of 0.04 L/min of pure argon was delivered to the growth chamber. During the deposition, the GST growth rate was monitored with an STM-2 rate monitor (INFICON Holding AG, Bad Ragaz, Switzerland), and the chamber pressure was in the range of high 10^−2^ mbar. The resulting GST deposition rate was around 0.72 nm/s.

### 2.2. Morphology Investigation

The morphological characterization of the GST layers was performed ex situ by means of an optical microscope (CARL ZEISS AG, Oberkochen, Germany) and an atomic force microscope (AFM) (PARK SYSTEMS Corp., Suwon, Korea) operating in noncontact mode.

### 2.3. X-ray Fluorescence (XRF)

Energy dispersive X-ray fluorescence (XRF) measurements were performed ex situ using a RIGAKU Nex DE VS spectrometer (APPLIED RIGAKU TECHNOLOGIES INC., Austin, TX, USA) equipped with a 60 kV X-ray tube and a silicon drift detector.

### 2.4. X-ray Diffraction (XRD)

XRD measurements were performed ex situ by a D8 Discover diffractometer (BRUKER, Billerica, MA, USA) equipped with a Cu X-ray source (Cu-Kα_1_ radiation λ = 1.5406 Å, 40 kV and 40 mA) and a DHS1100 dome-type heating stage (ANTON PAAR, Graz, Austria) for temperature measurements in a N_2_ atmosphere. Grazing incidence diffraction (GID) (ω–2θ) scans were acquired at different temperatures (T = 30–300 °C) during sample annealing. The average grain size of the grown GST layers was evaluated for selected diffraction peaks using the Scherrer equation [27]. The acquisition parameters were: incidence angle: 0.5°; 2θ steps: 0.08°; acquisition time: 25 min 17 s (30 °C), 25 min 22 s (130 °C), 25 min 22 s (140 °C), 1 h 16 min 7 s (200 °C), 1 h 17 min 7 s (300 °C), 1 h 29 min 59 s (PI substrate).

### 2.5. Raman

Raman spectra were acquired ex situ by means of a DXR2xi Raman imaging microscope (THERMOFISCHER, Waltham, MA, USA) equipped with a 532 nm laser source and a 50× objective. The Raman data acquisition was performed at RT in back-scattering geometry by using a 4 mW laser power at the sample surface.

### 2.6. Electrical Measurements

To characterize the GST electrical properties, the layer resistance R was measured in a probe station equipped with a model 2440 and two model 236 source meters (KEITHLEY, TEKTRONIX INC., Beaverton, OR, USA) The four-point collinear probe technique was used, and the volume resistivity was calculated as $\rho =\frac{\pi }{\ln2}\times R\times t\times R1$, where *t* is the film thickness, and *R1* is the Haldor Topsøe correction factor for thin samples of finite rectangular shape.

## 3. Results and Discussion

**Figure 1:**
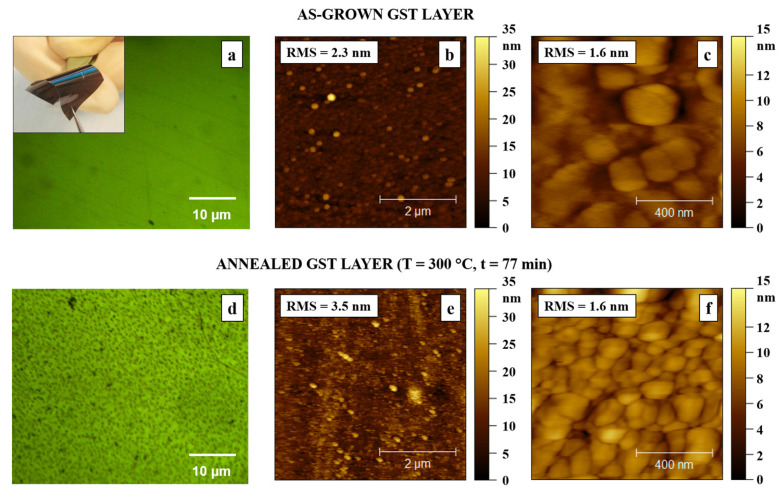
Ge_2_Sb_2_Te_5_ (GST) layers on polyimide (PI) substrate. As-grown: (**a**) optical microscope, (inset) photograph (1 × 1 cm) sample, (**b**) atomic force microscope (AFM) (5 × 5 µm), (**c**) AFM (1 × 1 µm). After annealing at 300 °C for 77 min: (**d**) optical microscope, (**e**) AFM (5 × 5 µm), and (**f**) AFM (1 × 1 µm).

Initially, we observed that the 140 nm thick GST layer (reasonable thickness for device realization, see explanation below), deposited on a 1 × 1 cm PI substrate, appeared uniform and continuous on a large scale (inset of Figure 1a). Considering a standard mushroom-type cell, the thickness of chalcogenide layers for PCM cells ranges from a few tens of nanometers to about 200 nm, depending on the heater size. High scalability is not expected for flexible substrates, for which the devices are on larger areas, rather than on rigid substrates; hence, thicker layers of the phase change materials are involved. According to the thin-film regime, thinner layers generally ensure better growth; nevertheless, thicker layers are preferable to observe possible defects such as exfoliation. Therefore, a thickness of 140 nm was selected as a good compromise. The presence of GST in our films was clearly distinguishable by the naked eye for their brown color, in contrast with the orange, transparent PI substrate.

The optical microscope data (Figure 1a) showed, on a smaller scale, that the as-grown GST layer was continuous and transparent. Even at a smaller scale, as presented in AFM observations (Figure 1b,c), the GST film surface was fairly uniform: few protrusions, but no scratches or deeps were found. The optical microscope image of the GST film, after annealing at 300 °C for 77 min (Figure 1d), revealed a different surface morphology, with the appearance of tiny spots, which will be subject to further investigation. The AFM images on 5 × 5 µm areas showed a surface roughness RMS value of 2.3 nm and 3.5 nm for the as-grown (Figure 1b) and the annealed GST layer (Figure 1e), respectively. Nevertheless, both the as-grown (Figure 1c) and annealed (Figure 1f) layers showed, on smaller areas of 1 × 1 µm, the same value of the surface roughness, namely RMS = 1.6 nm.

The XRF measurements confirmed that the composition of the grown GST layer was (Ge:Sb:Te = 2.01:1.97:5 within the 5% error), effectively reproducing the nominal composition of the sputtered target.

**Figure 2:**
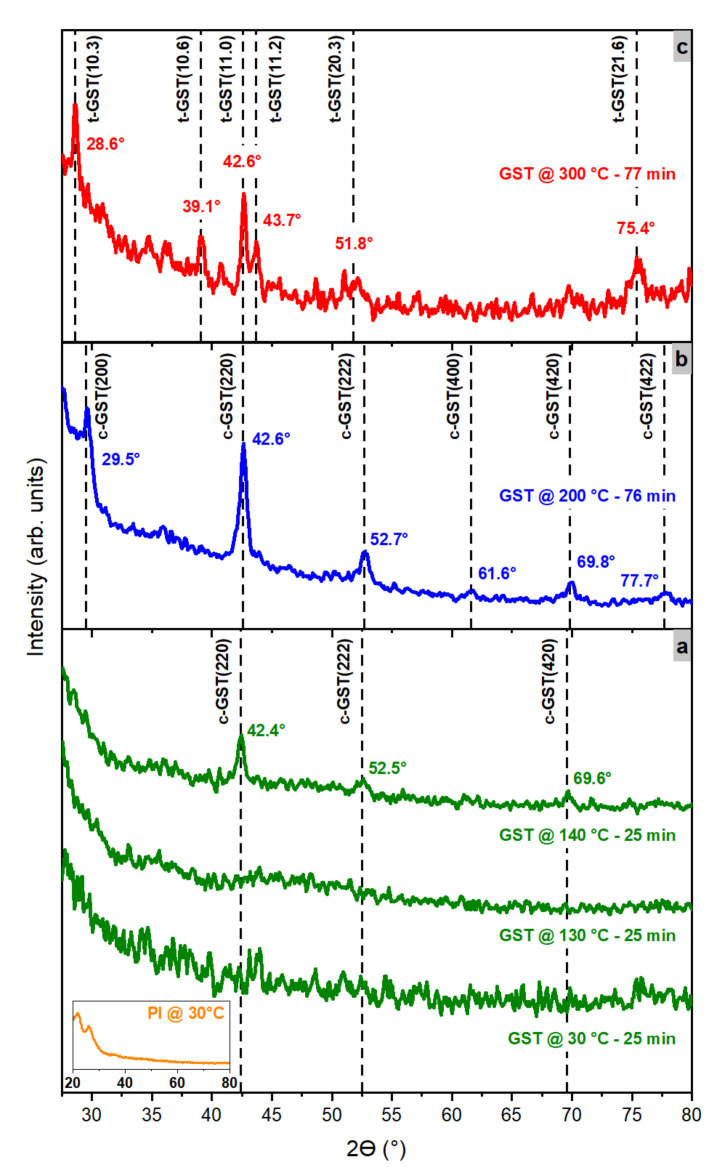
X-ray grazing incidence diffraction (GID) (ω–2θ) scans on PI/GST sample for (**a**) annealing experiment at T = 30, 130, and 140 °C (each temperature increase step was t = 25 min); (**b**) at T = 200 °C for t = 76 min; (**c**) at T = 300 °C for t = 77 min; (inset) of (**a**) X-ray GID (ω–2θ) scan on PI substrate at T = 30 °C acquired for t = 25 min. Dotted black lines indicate the positions of the experimental X-ray GID (ω–2θ) scan peaks.

Figure 2 shows the GID X-ray (ω–2θ) scans of the PI/GST layers during annealing under N_2_ atmosphere (T = 30–300 °C). The XRD diffractograms are displayed in the 2θ range of 27.5–80° to highlight the peaks of crystalline GST grains; the two broad peaks that characterized the clean PI substrate at ~21.8° and ~26° (inset of Figure 2a) were thus excluded.

The onset of the transition from amorphous (a-)GST to crystalline GST occurred when the temperature increased from 130 to 140 °C (Figure 2a): the peaks at 42.4°, 52.5°, and 69.6° were identified as the (220), (222), and (420) Bragg reflections of GST in the cubic (c-)GST crystalline structure, respectively. The XRD peak identification was performed by comparing the measured GID X-ray scans with those of previous experimental works [17,28] and diffractograms that were calculated with the cross-platform program VESTA [29,30,31]. Figure 2b shows the (ω–2θ) scan at T = 200 °C after annealing at T = 200 °C for 76 min: the peaks at 29.5°, 42.6°, 52.7°, 61.6°, 69.8°, and 77.7° were identified as the (200), (220), (222), (400), (420), and (422) Bragg reflections of c-GST, respectively. A comparison of the XRD peaks revealed that the crystallization of the amorphous GST had a preferential orientation along the [220] direction. The positions of the GST reflections are in very good agreement with the expected positions; therefore, strain was not present in our sample. The GST crystallization occurred through the polycrystalline relaxed grains.

The 2θ positions of the c-GST(220), c-GST(222) and c-GST (420) peaks shifted by 0.2° to higher values for the layer annealed at T = 200 °C for 77 min in comparison with the film annealed at T = 140 °C for twenty-five minutes. The XRD peak shifting toward larger 2θ values, with increasing the annealing temperature, was already observed by Lu et al. only for the c-GST(200) XRD reflection of 4 µm thick GST layers deposited by pulsed laser deposition on PI substrates [15]. The peak displacement was due to thermal lattice expansion, as the XRD graphs were acquired at different temperatures during annealing.

Figure 2c shows the (ω–2θ) scan at T = 300 °C after the annealing of a GST layer at T = 300 °C for 77 min: the peaks at 28.6°, 39.1°, 42.6°, 43.7°, 51.8°, and 75.4° were assigned to the (10.3), (10.6), (11.0), (11.2), (20.3), and (21.6) Bragg reflections of trigonal (t-)GST, respectively. The XRD data indicated that, after such annealing, the crystallization of a-GST into the t-GST crystalline structure had occurred.

The average crystalline grain size was 8.7 and 37.8 nm for the c-GST(200) and the t-GST(10.3) diffraction peak, respectively. This result is in agreement with the trend reported by Lu et al., who observed a linear increase in the average grain size from the c-GST(200) XRD reflection as a function of the annealing temperature [24]. This is an indication of grain growth during the ordering of the material upon annealing.

The PI substrate allows for an excellent thermal stability in the considered annealing temperatures, as it is well-known in the literature [32], implying no evident degradation of the GST layer.

**Figure 3:**
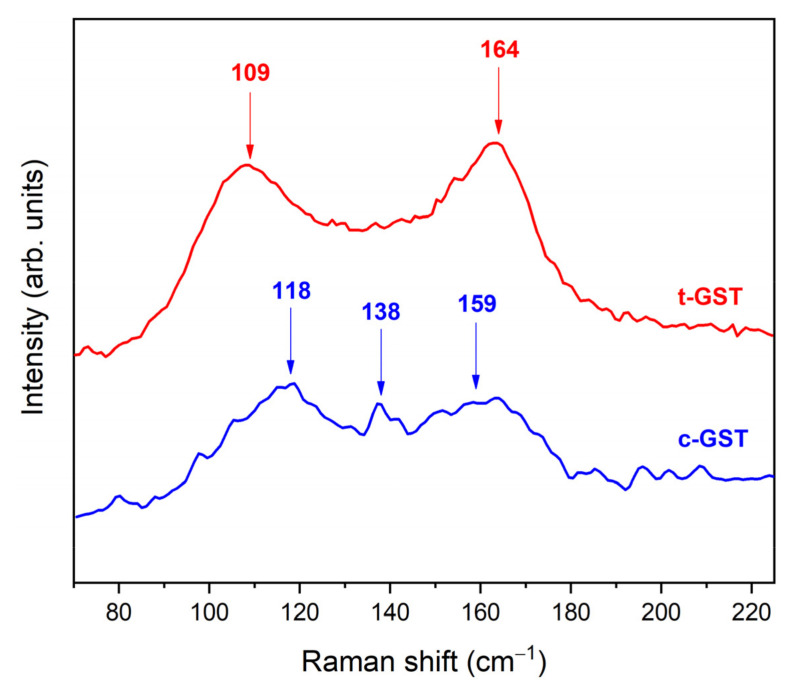
Raman spectra at room temperature (RT) of PI/GST sample after annealing at T = 200 °C for t = 76 min (blue curve) and after annealing at T = 300 °C for t = 77 min (red curve).

In Figure 3 the Raman spectra of c-GST and t-GST on PI are presented in a range from 75 to 225 cm^−1^. The PI substrate did not contribute to the Raman spectra in the range of interest (not shown here).

The spectrum of the c-GST layer shows three broad peaks centered at about 118, 138, and 159 cm^−1^. The peaks at 118 and 159 cm^−1^ were assigned to the E_g_ and A_1g_ modes of hexagonal Sb_2_Te_3_, respectively [33,34]. The peak at 138 cm^−1^ could be attributed to the A_1_ mode of corner-sharing GeTe_4-n_Ge_n_ (*n* = 1,2) tetrahedra [33] or, alternatively, to Te segregation in the sample [35]. The spectrum of the t-GST layer shows two main broad bands located at about 109 and 164 cm^−1^, which could be ascribed to the E- and A-type vibration modes, respectively [34].

The acquisition of the Raman spectra on PI substrates was not obvious because the relatively low thermal conductivity of the PI, which is in the range of 0.2 Wm^−1^ K^−1^ [36], is considered to be one of the major challenges for avoiding thermal failure in electronic devices. This feature limits the PI thermal management, especially in terms of heat dissipation; therefore, it must be taken into account in its response to laser heating. Regardless of whether infrared (wavelength 780 nm) or green (532 nm) laser light was used as the excitation source, we were not able to record a reliable Raman spectrum from the a-GST. Even at the lowest available power (0.1 mW) and with a very low exposure time (0.5 ms), we observed an immediate process of GST crystallization, which was highlighted by the formation of brighter spots with higher reflectivity on the sample surface. Therefore, we could not exclude that the observation of the peak at 138 cm^−1^ in the c-GST Raman spectrum might have been due to possible segregation of Te related to a modification induced by the laser light, causing local heating of the substrate [37].

**Figure 4:**
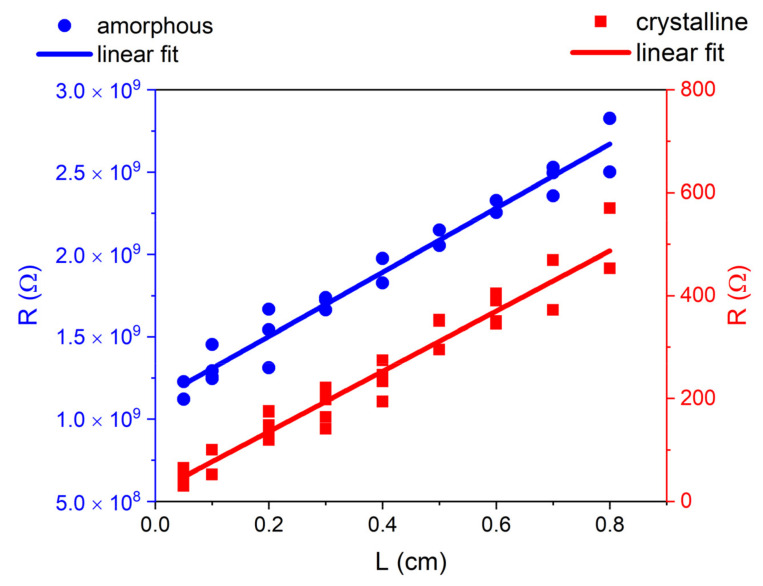
Resistance R at RT of the amorphous (as-deposited) and crystalline (after annealing at 300 °C for 77 min) GST films on PI, as a function of the contact distance L.

Several factors may affect the PCM’s electrical properties, such as film thickness, deposition method, and growth parameters. The electrical characteristics of the film are of interest as a direct indication for exploitation in memory devices of as-grown layers. A relevant contrast between the electrical resistance in the amorphous and crystalline states allows for a large on/off ratio in PCM cells. Hence, the amorphous (pristine as-deposited) and crystalline (after annealing) GST films on PI were electrically characterized at RT. In Figure 4, we report the linear dependence of the resistance R as a function of the contact distance L for amorphous and crystalline PI/GST.

The data showed the good homogeneity of the GST layers, which are thus compatible with large-area scalability. This confirmed the morphology results obtained by optical and AFM microscopy. For the crystalline and amorphous films, we measured a sheet resistance of R_sq, cryst_ = (332 ± 30) Ω/sq and R_sq, am_ = (2.6 ± 1.3) GΩ/sq, and a resistivity of *ρ*_cryst_ = (4.6 ± 0.4) mΩ·cm and *ρ*_am_ = (36 ± 18) kΩ·cm respectively. These values are in line with those of state-of-the-art GST films grown on rigid substrates [38,39,40,41], also deposited by other techniques such as atomic layer deposition [42] or molecular beam epitaxy [43]. The phase reached by the PCM in a cell, both for the set and reset states, is strongly dependent on the configuration, contacts, scaling level, and operating conditions. Therefore, in this study, fully amorphous and trigonal crystalline phases were considered as the most suitable to ensure a clear reference and an easy comparison with the values in the literature. The resistance ratio was separated into six orders of magnitude, namely R_am_/R_cryst_ ≈ 8 × 10^6^, which confirmed the excellent electrical features of the material deposited on the PI substrates. This is promising, because a key requirement of a PCM memory is that the programming window is large enough to avoid the possible ambiguity of data encoding and reduce resistance drift effects. In practical PCM devices, the resistance contrast is usually reduced by the contact and heater resistances; furthermore, the melt-quenched (thus amorphized) material has a lower resistivity with respect to the as-deposited amorphous phase. However, the large resistance ratio measured in our GST layers is in line with the values reported for materials suitable for PCM memories (around five orders of magnitude, [44]); thus, it is expected to allow for a large on/off ratio in flexible PCM memristors. Moreover, in a PCM cell, the high resistance contrast enables a gradual crystallization of an amorphous region by the application of repeated pulses. A controllable fractional variation of the resistance extends the possible applications to a multilevel domain. Gradual resistive switching is a key concept for analogue computing as it enables logic summation within a single PCM cell.

## 4. Conclusions

We deposited amorphous, 140 nm thick, Ge_2_Sb_2_Te_5_ films by RF-sputtering on flexible polyimide. The behavior of the films upon thermal annealing was studied during the amorphous-to-cubic and cubic-to-trigonal transitions. Both the as-grown and annealed layers were uniform and continuous with surface roughness RMS of 1.6 nm on 1 × 1 µm areas. GST layers of 140 nm can be grown in a fully amorphous phase on polyimide and the crystallization onset occurs at about 140 °C. Upon annealing, the transition from the amorphous to the cubic and trigonal phase was observed, as also confirmed by subsequent Raman characterization. Finally, the electrical characterization showed good homogeneity of the amorphous and crystalline trigonal GST layers, exhibiting a resistance ratio of R_am_/R_cryst_ ≈ 8 × 10^6^. This is a remarkable result in view of applications of PCM memristors for flexible smart devices devoted to IoT applications such as wearable and biocompatible sensors.

## Data Availability

Not applicable.
